# White Matter Hyperintensities Are Not Related to Symptomatology or Cognitive Functioning in Service Members with a Remote History of Traumatic Brain Injury

**DOI:** 10.1089/neur.2021.0002

**Published:** 2021-06-09

**Authors:** Sara M. Lippa, Kimbra Kenney, Gerard Riedy, John Ollinger

**Affiliations:** ^1^ National Intrepid Center of Excellence, Walter Reed National Military Medical Center, Bethesda, Maryland, USA.; ^2^ Department of Neurology, Uniformed Services University of the Health Sciences, Bethesda, Maryland, USA.

**Keywords:** cognition, military, symptoms, traumatic brain injury, white matter hyperintensities

## Abstract

This study aimed to determine whether magnetic resonance imaging (MRI) white matter hyperintensities (WMHs) are associated with symptom reporting and/or cognitive performance in 1202 active-duty service members with prior single or multiple mild traumatic brain injury (mTBI). Patients with mTBI evaluated at the National Intrepid Center of Excellence (NICoE) at Walter Reed National Military Medical Center (WRNMMC) were divided into those with (*n =* 632) and without (*n =* 570) WMHs. The groups were compared on several self-report scales including the Neurobehavioral Symptom Inventory (NSI), Post-Traumatic Stress Disorder (PTSD) Checklist-Civilian Version (PCL-C), Satisfaction with Life Scale (SWLS), and Short Form-36 Health Survey (SF-36). They were also compared on several neuropsychological measures, including tests of attention, working memory, learning and memory, executive functioning, and psychomotor functioning. After correction for multiple comparisons, there were no significant differences between the two groups on any self-reported symptom scale or cognitive test. When comparing a subgroup with the highest (20+) WMH burden (*n =* 60) with those with no WMHs (*n =* 60; matched on age, education, sex, race, rank, and TBI number), only SF-36 Health Change significantly differed between the subgroups; the multiple WMH subgroup reported worsening health over the past year (t[53] = 3.52, *p* = 0.001, *d* = 0.67) compared with the no WMH subgroup. These findings build on prior research suggesting total WMHs are not associated with significant changes in self-reported symptoms or cognitive performance in patients with a remote history of mTBI. As such, clinicians are encouraged to use caution when reporting such imaging findings.

## Introduction

White matter hyperintensities (WMHs) visible on fluid-attenuated inversion recovery (FLAIR) magnetic resonance imaging (MRI) are indicative of pathological findings including axonal/myelin degradation and gliosis.^1,2^ Although the presence of WMHs tends to increase in patients with a history of traumatic brain injury (TBI) relative to controls,^3^ WMHs are not specific for TBI.^4–6^ WMHs have been shown to increase with age,^7^ history of migraines,^8^ and vascular risk factors,^9^ and are identified even in some healthy children.^10^ When one or several WMHs are diagnosed in patients with a history of mild TBI (mTBI), the clinical utility of such imaging findings is unclear.

Prior studies of participants with a history of mTBI have found isolated and inconsistent relationships between WMH and cognition in relatively small samples.^11–13^ Clark and colleagues^11^ found an interaction between mTBI diagnosis and deep WMH volume on delayed memory, with patients with mTBI (*n =* 46) performing worse than controls (*n =* 22) as deep WMH volume increased. In contrast, there was no relationship between deep WMH volume and executive functioning, nor peri-ventricular WMH volume and learning/memory/executive functioning. Tate and associates^13^ demonstrated that within active-duty service members with a history of mTBI (*n =* 77), those with any WMH had worse working memory than those without WMH; however, they did not find any other differences on tests of processing speed, learning, and memory in this cohort. Spitz and colleagues^12^ found that patients with history of mild-severe TBI (*n =* 38) with high frontal WMH lesion load were slower to complete Trails B than patients with TBI with low frontal WMH lesion volume; however, there were no other differences between individuals with high and low total WMH volume or frontal WMH volume on the other 11 cognitive measures. Berginstrom and co-workers^14^ recently found no relationship between WMHs and cognition in patients with a history of mild-severe TBI (*n =* 59).

With regard to symptoms, one study found that as the number and size of WMHs increased, fatigue self-reports decreased.^14^ Other studies have found no relationship between WMHs and any self-reported psychological symptom.^11,13^

The relatively small sample sizes of the prior studies, combined with the lack of replication of results suggests that additional research is needed to clarify the relationship between WMHs and cognitive outcomes and chronic symptom burden following mTBI. The present study aimed to determine whether the presence of WMHs years after mTBI exposure is associated with current symptom reporting and cognitive performance in a large sample (*n =* 1202) of active-duty military service members with a history of mTBI. It also aimed to specifically determine whether individuals with the highest burden of WMHs (top 5% of the sample) reported increased symptomatology or evidenced reduced cognitive functioning compared with those with no WMHs.

## Methods

### Participants

Participants were 1202 U.S. military service members clinically evaluated 6 or more months following injury at the National Intrepid Center of Excellence (NICoE) at Walter Reed National Military Medical Center (WRNMMC) in Bethesda, Maryland, USA. Participants were prospectively enrolled after providing informed consent. Inclusion criteria for the overall study included Defense Enrollment Eligibility Reporting System eligibility. A history of major neurological or psychiatric conditions including psychosis, stroke, multiple sclerosis, or spinal cord injury was exclusionary for enrollment in the overall study. This research was approved by the Institutional Review Board of WRNMMC, is compliant with the Health Insurance Portability and Accountability Act, and was conducted in accordance with the Declaration of Helsinki guidelines.

Determination of mTBI history was based on medical record review of NICoE clinical assessments, which were often based on the patient's self-report. All participants had a diagnosis of mTBI in their record, and in many, but not all, cases, there was indication of the specific Veterans Affairs (VA)/Department of Defense (DoD) criteria^15^ that supported this diagnosis, including presence of loss of consciousness <30 min or presence of post-traumatic amnesia or alteration of consciousness <24 h.

Participants were selected from 2137 participants who underwent neuroimaging at NICoE and consented to the research protocol between August 2009 and March 2020. Participants were excluded if they did not complete any self-report measures (*n =* 249), did not have a clinical diagnosis of TBI (*n =* 380), or had a diagnosis of moderate, severe, or penetrating TBI (*n =* 79). Additionally, participants who did not complete the Neurobehavioral Symptom Inventory (NSI)^16^ within 35 days of their scan (*n =* 67), or who scored >22 on the Validity-10^17^ (*n =* 123) were excluded. Participants were also excluded if their scan had not been interpreted by the board-certified NICoE neuroradiologist (*n =* 28), or if their T2-FLAIR image quality was poor (e.g., due to motion, artifact, or technical problems; *n =* 9). This resulted in a final sample size of 1202. Additionally, for the analysis of cognitive data, we excluded 422 individuals who did not undergo neuropsychological testing within 35 days of their scan and 96 individuals who failed performance validity tests (PVTs; described below), for a final sample size of 684.

Participants were initially divided into two groups based on the presence/absence of WMHs: No WMHs (*n =* 570) and Any WHMs (*n =* 632). To investigate the most extreme participants, additional analyses were conducted by carefully matching the 60 participants with the highest (20+) WMH burden (i.e., those above the 95th percentile for WMHs in the sample) with those with No WMHs. Through sorting and inspection, individual matches were identified aiming for equal age, education, sex, race, rank, and number of TBIs. This resulted in two groups with no statistically significant difference on these matched variables (Table 1). For analysis of cognitive data, the same method was used to develop groups, with 312 participants with No WMHs and 372 participants with any number of identified WMHs. Additional analyses were conducted comparing the 35 participants with highest (20+) WMH burden and valid cognitive data with their matched pair with No WMHs.

**Table 1.** Demographic and Military Characteristics between Groups in The Total Sample and the Matched Pair Subsample

	Total sample					Matched pair subsample				
	No WMHs (*n* = 570)		Any WMHs (*n* = 632)			No WMHs (*n* = 60)		20+ WMHs (*n* = 60)		
	M	SD	M	SD	P	M	SD	M	SD	P
Age	35.5	7.7	39.0	7.5	<0.001	41.3	7.0	41.9	6.9	0.628
Years of education	14.3	2.2	14.4	2.2	0.336	14.8	2.3	14.5	2.1	0.548
	*Median*	*IQR*	*Median*	*IQR*	P	*Median*	*IQR*	*Median*	*IQR*	P
Number of TBIs	2	1-2	2	1-2	0.046^*^	2	1-2	2	1-2	0.718
	*N*	*%*	*N*	*%*	P^**^	*N*	*%*	*N*	*%*	P^**^
Men	556	97.5	609	96.4	0.236	59	98.3	59	98.3	1.00
Ethnicity					0.449					0.294
American Indian or Alaska Native	7	1.2	4	0.6	0.279	0	0.0	1	1.7	1.00
Asian or Pacific Islander	10	1.8	12	1.9	0.852	2	3.3	2	3.3	1.00
Black	11	1.9	19	2.9	0.232	3	5.0	4	6.7	1.00
Hispanic	10	1.8	19	3.0	0.158	1	1.7	1	1.7	1.00
White	376	66.0	425	67.4	0.638	45	75.0	37	61.7	0.116
Other	13	2.3	13	2.1	0.231	2	3.3	0	0.0	0.496
Unknown	143	25.1	140	22.2	0.237	7	11.7	15	25.0	0.059
Branch					0.001					0.351
Army	188	33.0	204	32.3	0.795	17	28.3	24	40.0	0.178
Air Force	48	8.4	39	6.2	0.133	6	10.0	3	5.0	0.491
Navy	249	43.7	322	51.0	0.012	35	58.3	29	48.3	0.272
Marines	84	14.7	66	10.4	0.024	2	3.3	4	6.7	0.679
Coast Guard	1	0.2	1	0.2	1.00	0	0.0	0	0.0	1.00
Rank					0.003			60		1.00
Cadet	5	0.9	2	0.3	0.266	0	0.0	0	0.0	1.00
E1-E4	61	10.7	38	6.0	0.003	1	1.7	1	1.7	1.00
E5-E9	383	67.2	451	71.6	0.117	42	70.0	42	70.0	1.00
O1-O3	42	7.4	28	4.4	0.036	3	5.0	3	5.0	1.00
O5-O7	58	10.2	79	12.5	0.205	8	13.3	9	15.0	0.793
Warrant Officer	21	3.7	33	5.2	0.199	6	10.0	5	8.3	0.752

^*^ The Any WMHs group had more TBIs than the No WMHs group.

^**^ Chi-square or Fisher's exact test for those with expected cell counts <5.

IQR, interquartile range; M, mean; SD, standard deviation; TBI, traumatic brain injury; WMH, white matter hyperintensity.

### MRI acquisition and coding

Anatomical MR images were obtained as part of an integrated protocol designed to examine various structural and functional aspects of TBI. Images were acquired on two 3T MRI units (Discovery 750; GE Healthcare, Milwaukee, WI, USA) with a 32-channel head coil (MR Instruments, Minneapolis, MN, USA). One unit was at the NICoE at the WRNMMC campus (April 2010 to March 2020) and the other was at Walter Reed Army Medical Center [WRAMC]; Washington, DC; (August 2009 to August 2011). The structural imaging included T1- and T2-weighted images, and T2-FLAIR images. Acquisition parameters were carefully optimized for high spatial resolution (small voxel size) with good contrast and short imaging times.

The structural MR images were collected in the sagittal plane with 1.2-mm slice-thickness and 0.6-mm overlap. The pre-contrast T1, T2, T2-FLAIR and post-contrast T1 and T2 images were reformatted into 3-mm sections at the axial and coronal orientations.

The anatomical imaging was transferred to a picture archiving and communication system (Agfa Healthcare, Mortsel, Belgium) for interpretation. The MR imaging studies were analyzed for the number of T2 hyperintensities by a single board-certified neuroradiologist (G.R.). There were no differences in the average number of WMHs between sites (WRNMMC total WMHs Med = 1; interquartile range [IQR] = 0–4; WRAMC total WMHs Med = 0; IQR = 0–1; *p* = 0.241).

### Measures

Self-report measures included the NSI^16^ total and cognitive, affective, vestibular, and somatosensory cluster scores^18^; the Post-Traumatic Stress Disorder (PTSD) Checklist (PCL)^19^ total and re-experiencing, avoidance, and hyperarousal cluster scores; the Satisfaction with Life Scale (SWLS)^20^; and the nine subscales from the Short Form-36 Health Survey (SF-36).^21^ The Validity-10 scale from the NSI was used to exclude individuals who may have been exaggerating symptoms (*n =* 123).^17^ Given the long timeline of this study, from August 2009 to March 2020, there was some variation in administration of self-report tests. In most cases, the PCL-C was administered. In 48 cases, the PCL-C was not completed, but the PCL-Military Version (PCL-M) was completed. The total and cluster scores of the PCL-C and PCL-M were treated as comparable. The PCL-M and PCL-C differ in that the PCL-M directs the participant to think about military trauma, whereas the PCL-C directs the participant to think about lifetime trauma; however, the item content is otherwise identical.

Neuropsychological test scores analyzed in the current study included the Test of Pre-morbid Functioning (TOPF);^22^ Wechsler Adult Intelligence Scale-IV (WAIS-IV)^23^ Verbal Comprehension Index (VCI), Perceptual Reasoning Index (PRI), Working Memory Index (WMI), Processing Speed Index (PSI); Wechsler Memory Scale-IV (WMS-IV)^24^ Logical Memory I and II; Trail Making Test^25^ A & B; California Verbal Learning Test (CVLT-II)^26^ Total Learning and Delayed Free Recall; Delis-Kaplan Executive Functioning System (DKEFS)^27^ Color Word Interference Test Word Reading and Inhibition, Tower Test Total Achievement, and Verbal Fluency Category Fluency and Letter Fluency; and Grooved Pegboard.^28^

Performance validity was measured with the Medical Symptom Validity Test (MSVT);^29^ Test of Memory Malingering (TOMM)^30^ Trial 1,^31^ Trial 2, and Retention Trial; Advanced Clinical Solutions Word Choice Test (WCT); and WAIS-IV Reliable Digit Span (RDS).^32^ Of participants who were administered any PVT (*n =* 780), most were administered the MSVT (*n =* 731) and RDS (*n =* 748). Additionally, 77 participants were administered the TOMM and 55 participants were administered the WCT. To ensure data were valid for the analysis of cognitive testing scores, participants must have been administered at least one and not failed any PVTs (*n =* 684). If participants who passed PVTs were missing data on some cognitive tests, they were excluded only from those relevant analyses.

### Statistical analysis

Descriptive statistics were calculated and group comparisons were conducted with analysis of variance (ANOVA), chi-square tests, and Mann-Whitney U tests. Effect sizes were computed with Cohen's *d* for continuous variables, *r* for non-parametric analyses (number of TBIs), and Cohen's *H* for binomial variables. For the comparison of self-reported symptoms between those with and without T2 WMHs, analyses of covariance (ANCOVAs) were conducted with age, education, and sex as covariates. For the comparison of cognitive performance between those with and without T2 WMHs, ANCOVAs were conducted with age, education, sex, pre-morbid intelligence (measured by TOPF Standard Score), and PCL-C total score as covariates.

Paired *t* tests were then conducted to assess differences in symptom reporting and cognitive performance between matched pair groups (No WMHs vs. 20+ WMHs). For the self-report analyses, each group had 60 participants. For the cognitive analyses, each group had 35 participants, as 20 of the 60 individuals with 20+ WMHs did not complete cognitive testing and 5 participants with 20+ WMHs failed PVTs. The relationship between number of total WMHs and outcomes was also evaluated through hierarchical linear regression, with the same covariates as in the ANCOVAs entered in the first step and total number of WMHs entered in the second step of the model. Given that this study was exploratory, the Benjamini-Hochberg False Discovery Rate (FDR)^33^ was used to keep the FDR at 0.05, correcting separately within each set of 19 self-report analyses and 18 cognitive analyses. As described by Benjamini and Hochberg,^33^ all *p*-values within a set of analyses are ranked from lowest to highest and compared against a new threshold *p*-value, generated from the following equation: $\frac{rank*alpha}{totalcomparisons}$. Working from highest to lowest *p*-values, actual *p*-values are compared against their respective threshold *p*-values. Once a *p*-value is identified that falls below the threshold value, all lower *p*-values are also considered significant. As opposed to a more stringent correction, such as the Bonferroni correction, which controls the family-wise error rate, or the chance of making any false-positive error, the FDR was chosen to control the proportion of false-positive errors made, limiting it to <5%.

For all models, missing covariate values were mean-imputed. In the analysis of self-reported symptoms, education was missing from 44 out of 1202 individuals. In the analysis of cognitive performance, 46 out of 684 individuals were missing TOPF Standard Score and 1 out of 684 participants was missing PCL-C data.

## Results

Just over half of the sample (*n =* 632, 52.6%) had at least one WMH on brain MRI exam. Those with WMHs had a median of 3 (IQR: 2–9) WMHs. Descriptive statistics and group comparisons for demographic and military characteristics are presented in Table 1. Those with WMHs were older (*p* < 0.001, Cohen's *d* = 0.47), had slightly more TBIs (*p* = 0.046, *r* = 0.06), were more likely to be in the Navy (*p* = 0.012, Cohen's *H* = 0.15), less likely to be in the Marines (*p* = 0.024, *H* = 0.12), and were less likely to have a rank of E1–E4 (*p* = 0.003, *H* = 0.17) or O1–O3 (*p* = 0.036, *H* = 0.13). There were no statistical differences in education or ethnicity between the groups.

### White matter hyperintensities and self-reported symptoms

Those with WMHs reported a worse change in health over the past year compared with those without WMHs (F[1,1140] = 5.05, *p* = 0.025); however, this result did not survive the FDR correction. There were no differences between WMH groups on any other self-report scales or subscales (see Table 2). Hierarchical regression similarly revealed that total number of WMHs was related only to worse reported health change over the past year (R^2^Δ = 0.005, β = −0.074, *p* = 0.012); however, this also did not survive the FDR correction.

**Table 2.** Self-Reported Symptom Means, Standard Errors, and Comparison between Those with and without WMH, Adjusted for Age, Education, and Sex

	No WMH (*n* = 570)		Any WMH (*n* = 632)				
	M	SE	M	SE	*F*	*P*	Ƞ_p_^2^
NSI Total	33.1	0.6	33.2	0.6	0.050	0.823	0.000
NSI Somatosensory	8.0	0.2	8.2	0.2	0.209	0.647	0.000
NSI Cognitive	8.0	0.2	8.1	0.1	0.054	0.816	0.000
NSI Affective	11.7	0.2	11.7	0.2	0.000	0.995	0.000
NSI Vestibular	2.8	0.1	2.8	0.1	0.126	0.722	0.000
PCL-C Total	44.0	0.7	44.5	0.6	0.217	0.641	0.000
PCL-C Reexperiencing	11.0	0.2	11.3	0.2	0.999	0.318	0.001
PCL-C Avoidance	17.4	0.3	17.5	0.3	0.026	0.873	0.000
PCL-C Hyperarousal	15.6	0.2	15.7	0.2	0.048	0.826	0.000
SWLS	22.4	0.4	22.5	0.3	0.044	0.834	0.000
SF-36 Physical Functioning	74.7	0.9	74.5	0.9	0.010	0.921	0.000
SF-36 Role Limitations due to Physical Health	38.1	1.7	36.4	1.6	0.555	0.457	0.000
SF-36 Role Limitations due to Emotional Problems	48.3	1.9	48.1	1.8	0.008	0.928	0.000
SF-36 Energy/Fatigue	31.2	0.9	32.2	0.9	0.647	0.421	0.001
SF-36 Emotional Well-Being	57.0	0.9	57.2	0.8	0.021	0.884	0.000
SF-36 Social Functioning	56.0	1.1	55.3	1.1	0.219	0.640	0.000
SF-36 Pain	57.9	0.9	56.6	0.8	1.056	0.304	0.001
SF-36 General Health	59.0	0.9	59.9	0.8	0.515	0.473	0.000
SF-36 Health Change	45.0	1.1	41.7	1.0	5.054	0.025	0.004

*P*-values are prior to Benjamini-Hochberg False Discovery Rate correction. No *p*-values survived this correction.

M, mean; NSI, Neurobehavioral Symptom Inventory; PCL-C, Post-Traumatic Stress Disorder Checklist-Civilian Version; SE, standard error of the mean; SF-36, Short Form-36 Health Survey; SWLS, Satisfaction with Life Scale; WMH, white matter hyperintensity.

When comparing patients with a remote history of mTBI with highest (20+) WMH burden (*n* = 60) with a group with No WMHs (*n* = 60) who were matched on age, education, sex, race, rank, and number of TBIs, PCL-C avoidance (t[59] = −2.28, *p* = 0.027, *d* = 0.34), SF-36 Social Functioning (t[53] = 2.23, *p* = 0.030, *d* = 0.37), SF-36 General Health (t[53] = 2.20, *p* = 0.032, *d* = 0.38), and SF-36 Health Change (t[53] = 3.52, *p* = 0.001, *d* = 0.67) were significantly different between the groups, with those with 20+ WMHs reporting more symptomatology. After the Benjamini-Hochberg correction, only the SF-36 Health Change single question remained significant, suggesting those with the highest WMH burden reported worsening in their overall health over the past year compared to those with No WMHs (see Table 3).

**Table 3.** Matched Pair Analyses of Self-Report Data

	No WMH**(*n* = 60)		20+ WMH**(*n* = 60)				
	M	SD	M	SD	*t*	*P*	*d*
NSI Total	30.5	12.7	34.6	12.6	-1.79	0.078	0.32
NSI Somatosensory	7.1	3.6	8.0	4.1	-1.38	0.172	0.23
NSI Cognitive	7.5	3.7	8.4	3.4	-1.33	0.189	0.25
NSI Affective	11.2	4.6	12.5	5.0	-1.50	0.138	0.27
NSI Vestibular	2.4	2.0	2.7	1.8	-0.73	0.468	0.16
PCL-C Total	40.5	13.5	45.0	15.5	-1.99	0.052	0.31
PCL-C Reexperiencing	9.8	4.5	11.0	5.1	-1.51	0.138	0.24
PCL-C Avoidance	15.6	6.5	17.9	7.1	-2.28	0.027	0.34
PCL-C Hyperarousal	15.1	4.7	16.1	4.8	-1.12	0.266	0.20
SWLS	24.2	6.0	21.5	7.7	2.00	0.056	0.39
SF-36 Physical Functioning	77.6	20.2	75.2	18.4	0.70	0.485	0.12
SF-36 Role Limitations due to Physical Health	37.3	39.1	36.3	38.8	0.13	0.896	0.03
SF-36 Role Limitations due to Emotional Problems	52.8	44.1	45.3	45.3	0.88	0.381	0.17
SF-36 Energy/Fatigue	30.3	19.5	30.6	20.2	-0.07	.942	0.02
SF-36 Emotional Well-Being	59.7	18.7	55.9	21.4	1.15	.256	0.19
SF-36 Social Functioning	61.6	26.5	51.9	25.4	2.23	.030	0.37
SF-36 Pain	57.7	19.6	54.3	17.5	0.94	.353	0.18
SF-36 General Health	63.2	18.9	56.0	19.5	2.20	.032	0.38
SF-36 Health Change	51.4	23.0	36.6	21.0	3.52	.001	0.67

*P*-values are prior to Benjamini-Hochberg False Discovery Rate correction. Only SF-36 Health Change survived this correction.

M, mean; NSI, Neurobehavioral Symptom Inventory; PCL-C, Post-Traumatic Stress Disorder Checklist-Civilian Version; SF-36, Short Form-36 Health Survey; SWLS, Satisfaction with Life Scale; WMH, white matter hyperintensity.

### White matter hyperintensities and neuropsychological test performance

Those with WMHs performed worse on the WAIS-IV PSI (F[1,665] = 5.278, *p* = 0.022) and VCI (F[1,615] = 4.496, *p* = 0.034), as well as Logical Memory Delayed Recall (F[1,615] = 6.485, *p* = 0.011) and CVLT-II Total Learning (F[1,638] = 6.371, *p* = 0.012). However, these results also did not survive the FDR correction. There were no differences between WMH groups on any other cognitive measures (see Table 4). Hierarchical regression revealed that total number of WMHs was related only to worse performance on WAIS-IV PSI (R^2^Δ = 0.011, β = −0.106, *p* = 0.007) and non-dominant-hand grooved pegboard (R^2^Δ = 0.007, β = −0.084, *p* = 0.037); however, these also did not survive the FDR correction.

**Table 4.** Neuropsychological Test Normative Score Means, Standard Errors, and Comparison between Those with and without WMH, Adjusted for Age, Education, and Sex, Pre-morbid Intelligence, and PTSD Symptom Severity

	No WMH (*n* = 312)		Any WMH (*n* = 372)				
	M	SE	M	SE	*F*	*P*	Ƞ_p_^2^
TOPF SS	101.1	0.5	102.2	0.5	2.04	.154	.003
PSI SS	105.3	0.7	103.0	0.7	5.28	.022	.008
WMI SS	106.6	0.6	105.8	0.6	0.76	.384	.001
VCI SS	111.3	0.5	109.8	0.5	4.5	.034	.007
PRI SS	112.6	0.7	112.4	0.6	0.08	.773	.000
Logical Memory II ss	11.1	0.2	10.5	0.2	6.49	.011	.010
Logical Memory I ss	11.5	0.1	11.2	0.1	2.38	.123	.004
TMT A T-score	53.7	0.6	53.4	0.6	0.12	.725	.000
TMT B T-score	51.6	0.6	51.9	0.5	0.11	.742	.000
CVLT-II LDFR z-score	0.36	0.06	0.27	0.05	1.07	.303	.002
CVLT-II Trials 1-5 T-score	56.5	0.6	54.5	0.5	6.37	.012	.010
DKEFS CWIT Inhibition ss	11.0	0.2	10.7	0.2	1.02	.312	.002
DKEFS CWIT Word Reading ss	10.8	0.1	10.9	0.1	0.35	.554	.001
DKEFS Tower Total Achievement ss	12.1	0.2	12.0	0.1	0.64	.426	.001
DKEFS Category Fluency ss	12.8	0.2	12.7	0.2	0.02	.881	.000
DKEFS Letter Fluency ss	11.5	0.2	11.3	0.2	0.76	.383	.001
GP Dominant T-score	53.1	0.6	52.1	0.5	1.82	.178	.003
GP Non-dominant T-score	53.2	0.6	52.2	0.5	1.34	.247	.002

*P*-values are prior to Benjamini-Hochberg False Discovery Rate correction. No *p*-values survived this correction.

CVLT-II, California Verbal Learning Test-Second Edition; CWIT, Color-Word Interference Test; DKEFS, Delis-Kaplan Executive Functioning System; GP, Grooved Pegboard; LDFR, long delay free recall; M, mean; PRI, Perceptual Reasoning Index; PSI, Processing Speed Index; PTSD, post-traumatic stress disorder; SE, standard error of the mean; ss, scaled score; SS, standard score; TMT, Trail Making Test; TOPF, Test of Pre-morbid Functioning; VCI, Verbal Comprehension Index; WMH, white matter hyperintensity; WMI, Working Memory Index

When comparing those with 20+ WMHs with a matched group of those with No WMHs, there were no differences in cognitive performance on any neuropsychological test included in the battery between the groups (see Table 5).

**Table 5.** Matched Pair *t* Tests Comparing Those with 20+ WMHs and Those with no WMHs on Neuropsychological Tests

	No WMH (*n* = 35)		20+ WMH (*n* = 35)				
	M	SD	M	SD	*t*	*P*	*d*
TOPF SS	101.9	8.9	104.5	9.2	-1.25	0.220	0.29
PSI SS	105.5	12.5	101.9	11.4	1.56	0.128	0.30
WMI SS	104.3	10.5	108.6	15.8	-1.40	0.169	0.33
VCI SS	113.5	12.4	112.4	10.7	0.40	0.692	0.10
PRI SS	110.6	14.9	116.1	11.2	-1.87	0.070	0.42
Logical Memory II ss	11.8	3.0	10.8	2.3	1.76	0.088	0.38
Logical Memory I ss	12.1	2.7	11.2	2.3	1.99	0.055	0.36
TMT A T-score	54.3	9.9	53.3	10.6	0.47	0.638	0.10
TMT B T-score	52.4	9.0	52.8	10.1	-0.18	0.856	0.04
CVLT-II LDFR z score	0.47	0.90	0.47	0.80	0.00	1.00	0.00
CVLT-II Trials 1-5 T-score	57.5	8.5	57.4	9.6	0.07	0.941	0.01
DKEFS CWIT Inhibition ss	10.7	2.8	10.9	2.4	-0.28	0.782	0.08
DKEFS CWIT Word Reading ss	11.0	2.0	10.6	2.0	0.80	0.430	0.20
DKEFS Tower Total Achievement ss	11.6	2.4	12.7	2.9	-1.41	0.172	0.42
DKEFS Category Fluency ss	12.9	3.6	13.1	0.6	-0.29	0.777	0.10
DKEFS Letter Fluency ss	11.5	3.0	12.2	3.4	-0.98	0.333	0.22
GP Dominant T-score	51.5	9.4	53.6	8.8	-1.05	0.303	0.23
GP Non-dominant T-score	51.6	9.5	50.8	11.2	0.34	0.735	0.08

*P*-values are prior to Benjamini-Hochberg False Discovery Rate correction. No *p*-values survived this correction.

CVLT-II, California Verbal Learning Test-Second Edition; CWIT, Color-Word Interference Test; DKEFS, Delis-Kaplan Executive Functioning System; GP, Grooved Pegboard; LDFR, long delay free recall; M, mean; PRI, Perceptual Reasoning Index; PSI, Processing Speed Index; SD, standard deviation; ss, scaled score; SS, standard score; TMT, Trail Making Test; TOPF, Test of Pre-morbid Functioning; VCI, Verbal Comprehension Index; WMH, white matter hyperintensity; WMI, Working Memory Index.

## Discussion

This study sought to determine whether whole-brain counts of WMHs correspond with increased neurobehavioral symptomatology or cognitive dysfunction in a sample of service members referred for treatment of a remote history of mTBI. Notably, of 37 self-report and cognitive measures across three sets of analyses, there was no association with WMHs on 36 of these measures after correction for multiple comparisons. The only significant finding was that those with 20+ WMHs reported a greater decline in health over the past year than matched individuals with No WMHs. This effect was medium-large (Cohen's *d* = 0.67).

This cross-sectional study cannot speak to the causal direction of the relationship between self-reported deterioration in health status over the past year and WMHs. Importantly, WMHs are largely non-specific, can occur in a variety of medical conditions, and are often observed in healthy people. Although they may be the result of TBI, they are also present in apparently healthy individuals and increase with age, and appear also in individuals with migraines and cerebrovascular risk factors. The sole significant relationship found in this analysis derived from a survey question that asked about subjective worsening in general health over the past year. Because the majority of patients were referred to NICoE several years after their injury, it seems unlikely that WMHs resulting from TBI are influencing the recent health decline. Rather, other systemic etiologies, such as advancing age and/or associated comorbidities (e.g., hypertension, diabetes, migraine) that developed or progressed more recently may be contributing to increased WMHs. However, future longitudinal investigation with serial imaging will be necessary to confirm the direction of this relationship and clarify the timing of the appearance of the WMH in relation to a TBI and other medical conditions.

The absence of a relationship between WMHs and both self-reported symptoms and objective cognitive performance suggests that WMHs are not critical to understanding the clinical presentation of patients with a history of mTBI. On the other hand, given that in older individuals WMHs are often an indicator of cardiovascular disease^34^ and are associated with increased risk of stroke^35^ and death,^35,36^ a large number of WMHs may warrant additional medical evaluation and treatment for long-term cerebrovascular health promotion.^37^ It is also possible that a large number of WMHs is more detrimental as one ages, and that although younger individuals, as in our cohort, are able to compensate for WMHs, there may be a more measureable detrimental effect in older individuals. Indeed, our findings stand in contrast to much of the extant literature investigating the relationship between WMHs and cognition in samples of older individuals who have not necessarily sustained a TBI in the past. Meta-analyses have shown that WMHs are associated with cognitive decline in healthy older adults,^38^ as well as in individuals diagnosed with mild cognitive impairment and dementia.^39^

Nevertheless, any discussion of WMHs with patients with a history of TBI should be careful to avoid misattribution of ongoing observed or reported difficulties to WMHs. For instance, if WMHs are presented as evidence of mTBI and/or a sign of brain damage, patients may misunderstand and conclude that their symptoms are permanent, potentially impeding their recovery. To minimize iatrogenesis, it should be emphasized that WMHs are non-specific, frequently occur in apparently healthy individuals, and are unrelated to both self-assessed symptoms and cognitive performance, even in individuals with 20+ WMHs. The lack of relationship between WMHs and a large variety of measures of cognition and self-reported symptoms in this large sample of service members with a history of TBI should be considered when discussing prognoses with individuals with a history of mTBI and WMHs.

Limitations of the present study include that only one board-certified neuroradiologist reviewed each set of scans, which may have resulted in a biased count of WMHs. On the other hand, this is also advantageous as it ensured that all scans were reviewed by the same neuroradiologist, removing any potential inter-rater reliability issues. Additionally, mTBI diagnosis was determined from the medical record and the quality of the information regarding TBI history varied, precluding a detailed characterization of the sample in this regard. Further, the TBI diagnosis was often based on self-reported symptoms years and even decades after injury, rather than medical records obtained at the time of acute injury.

Unfortunately, oftentimes records from the injury event itself are not maintained or readily accessible and this is a limitation of most retrospective studies of remote TBI.^40^ Recently, the Ohio State University TBI Questionnaire^41,42^ was administered to a cohort of NICoE participants in person and final determination of TBI history was determined through consensus conference. In this recent cohort >80% were diagnosed with TBI, which is similar to the rate of TBI diagnosis in this analysis. Additionally, this study did not include a control group of participants without TBI, as cognitive data were collected clinically with no corresponding data collection in an appropriate control group. Finally, this study did not have reliable access to other medical conditions of interest that are associated with an increased risk of WMHs and/or reduced cognitive functioning (e.g., vascular risk factors, migraines).

Despite these limitations, this study has several strengths, including the largest sample of participants with mTBI to-date and removal of participants with questionable symptom validity and/or performance validity, which have been shown to greatly impact self-report^43^ and cognitive test data,^44^ respectively.

## Conclusion

This study investigated the relationship of WMHs and self-reported symptoms and cognition in active duty service members with a history of one or more mTBIs. The findings build on past research that, in aggregate, suggests WMHs are not meaningfully related to neurobehavioral symptoms, physical functioning, or cognitive performance in patients with a remote history of mTBI. As such, clinicians are encouraged to use caution when reporting such imaging findings. Future longitudinal research should continue to investigate the relationship between vascular risk factors and long-term outcomes following TBI.

## Abbreviations Used

ANCOVA: analysis of covariance
ANOVA: analysis of variance
CLVT-II: California Verbal Learning Test
DKEFS: Delis-Kaplan Executive Functioning System
DoD: Department of Defense
FDR: Benjamini-Hochberg False Discovery Rate
FLAIR: fluid-attenuated inversion recovery
IQR: interquartile range
MRI: magnetic resonance imaging
MSVT: Medical Symptom Validity Test
mTBI: mild traumatic brain injury
NICoE: National Intrepid Center of Excellence
NSI: Neurobehavioral Symptom Inventory
PCL: Post-Traumatic Stress Disorder Checklist
PCL-C: Post-Traumatic Stress Disorder Checklist-Civilian Version
PCL-M: Post-Traumatic Stress Disorder Checklist-Military Version
PRI: Perceptual Reasoning Index
PSI: Processing Speed Index
PTSD: post-traumatic stress disorder
PVT: performance validity test
RDS: Wechsler Adult Intelligence Scale-IV Reliable Digit Span
SD: standard deviation
SE: standard error of the mean
SF-36: Short Form-36 Health Survey
SWLS: Satisfaction with Life Scale
TBI: traumatic brain injury
TOMM: Test of Memory Malingering
TOPF: Test of Premorbid Functioning
VA: Veterans Affairs
VCI: Verbal Comprehension Index
WAIS-IV: Wechsler Adult Intelligence Scale-IV
WCT: Advanced Clinical Solutions Word Choice Test
WMH: white matter hyperintensity
WMI: Working Memory Index
WMS-IV: Wechsler Memory Scale-IV
WRAMC: Walter Reed Army Medical Center
WRNMMC: Walter Reed National Military Medical Center

## References

[1] Fazekas, F., Kleinert, R., Offenbacher, H., Schmidt, R., Kleinert, G., Payer, F., Radner, H., and Lechner, H. (1993). Pathologic correlates of incidental MRI white matter signal hyperintensities. Neurology 43, 1683–1689 8414012 10.1212/wnl.43.9.1683

[2] Pantoni, L., and Garcia, J.H. (1997). Pathogenesis of leukoaraiosis: a review. Stroke 28, 652–659 9056627 10.1161/01.str.28.3.652

[3] Trifan, G., Gattu, R., Haacke, E.M., Kou, Z., and Benson, R.R. (2017). MR imaging findings in mild traumatic brain injury with persistent neurological impairment. Magn. Reson. Imaging 37, 243–251 27939436 10.1016/j.mri.2016.12.009

[4] Bigler, E.D. (2013). Neuroimaging biomarkers in mild traumatic brain injury (mTBI). Neuropsychol. Rev. 23, 169–209 23974873 10.1007/s11065-013-9237-2

[5] Hopkins, R.O., Beck, C.J., Burnett, D.L., Weaver, L.K., Victoroff, J., and Bigler, E.D. (2006). Prevalence of white matter hyperintensities in a young healthy population. J. Neuroimaging 16, 243–251 16808826 10.1111/j.1552-6569.2006.00047.x

[6] Iverson, G.L., Hakulinen, U., Waljas, M., Dastidar, P., Lange, R.T., Soimakallio, S., and Ohman, J. (2011). To exclude or not to exclude: white matter hyperintensities in diffusion tensor imaging research. Brain Inj. 25, 1325–1332 22077537 10.3109/02699052.2011.608409

[7] Morris, Z., Whiteley, W.N., Longstreth, W.T. Jr., Weber, F., Lee, Y.C., Tsushima, Y., Alphs, H., Ladd, S.C., Warlow, C., Wardlaw, J.M., and Al-Shahi Salman, R. (2009). Incidental findings on brain magnetic resonance imaging: systematic review and meta-analysis. BMJ 339, b3016 19687093 10.1136/bmj.b3016 PMC2728201

[8] Kruit, M.C., van Buchem, M.A., Hofman, P.A., Bakkers, J.T., Terwindt, G.M., Ferrari, M.D., and Launer, L.J. (2004). Migraine as a risk factor for subclinical brain lesions. JAMA 291, 427–434 14747499 10.1001/jama.291.4.427

[9] Wardlaw, J.M., Valdés Hernández, M.C., and Muñoz-Maniega, S. (2015). What are white matter hyperintensities made of? Relevance to vascular cognitive impairment. J. Am. Heart Assoc. 4, 001140 26104658 10.1161/JAHA.114.001140 PMC4599520

[10] Bigler, E.D., Deibert, E., and Filley, C.M. (2013). When is a concussion no longer a concussion? Neurology 81, 14–15 23709592 10.1212/WNL.0b013e318299cd0e

[11] Clark, A.L., Sorg, S.F., Schiehser, D.M., Luc, N., Bondi, M.W., Sanderson, M., Werhane, M.L., and Delano-Wood, L. (2016). Deep white matter hyperintensities affect verbal memory independent of PTSD symptoms in veterans with mild traumatic brain injury. Brain Inj. 30, 864–871 27058006 10.3109/02699052.2016.1144894

[12] Spitz, G., Maller, J.J., Ng, A., O'Sullivan, R., Ferris, N.J., and Ponsford, J.L. (2013). Detecting lesions after traumatic brain injury using susceptibility weighted imaging: a comparison with fluid-attenuated inversion recovery and correlation with clinical outcome. J. Neurotrauma 30, 2038–2050 23952803 10.1089/neu.2013.3021

[13] Tate, D.F., Gusman, M., Kini, J., Reid, M., Velez, C.S., Drennon, A.M., Cooper, D.B., Kennedy, J.E., Bowles, A.O., Bigler, E.D., Lewis, J.D., Ritter, J., and York, G.E. (2017). Susceptibility weighted imaging and white matter abnormality findings in service members with persistent cognitive symptoms following mild traumatic brain injury. Mil. Med. 182, e1651–e1658 28290939 10.7205/MILMED-D-16-00132

[14] Berginstrom, N., Nordstrom, P., Nyberg, L., and Nordstrom, A. (2020). White matter hyperintensities increases with traumatic brain injury severity: associations to neuropsychological performance and fatigue. Brain Inj. 34, 415–420 32037894 10.1080/02699052.2020.1725124

[15] Department of Veterans Affairs/Department of Defense. (2016). VA/DOD clinical practice guideline for the management of concussion-mild traumatic brain injury. https://www.healthquality.va.gov/guidelines/MH/mdd/VADoDMDDCPGFINAL82916.pdf (Last accessed 41, 2021).

[16] Cicerone, K.D., and Kalmar, K. (1995). Persistent postconcussion syndrome: the structure of subjective complaints after mild traumatic brain injury. J. Head Trauma Rehabil. 10, 1–17

[17] Vanderploeg, R.D., Cooper, D.B., Belanger, H.G., Donnell, A.J., Kennedy, J.E., Hopewell, C.A., and Scott, S.G. (2014). Screening for postdeployment conditions: development and cross-validation of an embedded validity scale in the neurobehavioral symptom inventory. J. Head Trauma Rehabil. 29, 1–10 23474880 10.1097/HTR.0b013e318281966e

[18] Vanderploeg, R.D., Silva, M.A., Soble, J.R., Curtiss, G., Belanger, H.G., Donnell, A.J., and Scott, S.G. (2015). The structure of postconcussion symptoms on the Neurobehavioral Symptom Inventory: a comparison of alternative models. J. Head Trauma Rehabil. 30, 1–11 24263177 10.1097/HTR.0000000000000009

[19] Weathers, F.W., Litz, B.T., Huska, J.A., and Keane, T.M. (1994). PTSD Checklist-Civilian Version. National Center for PTSD, Behavioral Sciences Division: Boston, MA

[20] Corrigan, J. (2013). The Satisfaction with Life Scale. The Center for Outcome Measurement in Brain Injury. http://www.tbims.org/combi/swls/index.html (Last accessed 41, 2021)

[21] Ware, J.E., Snow, K.K., Kosinski, M., and Gandek, B. (1993). SF-36 Health Survey: Manual and Interpretation Guide. The Health Institute, New England Medical Center: Boston, MA

[22] Pearson. (2009). Advanced Clinical Solutions for Use with WAIS-IV and WMS-IV. Pearson: San Antonio, TX

[23] Wechsler, D. (2008). Wechsler Adult Intelligence Scale-Fourth Edition: Technical and Interpretive Manual. Pearson: San Antonio, TX

[24] Wechsler, D. (2009). Wechsler Memory Scale-Fourth Edition: Technical and Interpretive Manual. Pearson: San Antonio, TX

[25] Reitan, R.M. (1958). Validity of the Trail Making Test as an indicator of organic brain damage. Percept. Mot. Skills 8, 271–276

[26] Delis, D.C., Kramer, J.H., Kaplan, E., and Ober, B.A. (2000). California Verbal Learning Test. The Psychological Corporation: San Antonio, TX

[27] Delis, D.C., Kaplan, E., and Kramer, J.H. (2001). Delis-Kaplan Executive Functioning System (D-KEFS): Examiner's Manual. The Psychological Corporation: San Antonio, TX

[28] Matthews, C.G., and Klove, K. (1964). Instruction Manual for the Adult Neuropsychology Test Battery. University of Wisconsin Medical School: Madison, WI

[29] Green, P. (2004). Green's Medical Symptom Validity Test (MSVT) for Microsoft Windows: User's Manual. Green's Publishing: Edmonton, Canada

[30] Tombaugh, T.N. (1996). TOMM: Test of Memory Malingering. Multi-Health Systems: Tonawanda, NY

[31] Denning, J.H. (2012). The efficiency and accuracy of the Test of Memory Malingering trial 1, errors on the first 10 items of the test of memory malingering, and five embedded measures in predicting invalid test performance. Arch. Clin. Neuropsychol. 27, 417–432 22543569 10.1093/arclin/acs044

[32] Axelrod, B.N., Fichtenberg, N.L., Millis, S.R., and Wertheimer, J.C. (2006). Detecting incomplete effort with Digit Span from the Wechsler Adult Intelligence Scale-Third Edition. Clin. Neuropsychol. 20, 513–523 16895862 10.1080/13854040590967117

[33] Benjamini, Y., and Hochberg, Y. (1995). Controlling the false discovery rate: a practical and powerful approach to multiple testing. J. R. Stat. Soc. Ser. B. Stat. Methodol. 57, 289–300

[34] Moroni, F., Ammirati, E., Rocca, M.A., Filippi, M., Magnoni, M., and Camici, P.G. (2018). Cardiovascular disease and brain health: focus on white matter hyperintensities. Int. J. Cardiol. Heart Vasc. 19, 63–69 29946567 10.1016/j.ijcha.2018.04.006 PMC6016077

[35] Debette, S., and Markus, H.S. (2010). The clinical importance of white matter hyperintensities on brain magnetic resonance imaging: systematic review and meta-analysis. BMJ 341, c3666 20660506 10.1136/bmj.c3666 PMC2910261

[36] Hasan, T.F., Barrett, K.M., Brott, T.G., Badi, M.K., Lesser, E.R., Hodge, D.O., and Meschia, J.F. (2019). Severity of white matter hyperintensities and effects on all-cause mortality in the Mayo Clinic Florida Familial Cerebrovascular Diseases Registry. Mayo Clin. Proc. 94, 408–416 30832790 10.1016/j.mayocp.2018.10.024

[37] Merino, J.G. (2019). White matter hyperintensities on magnetic resonance imaging: what is a clinician to do? Mayo Clin. Proc. 94, 380–382 30832786 10.1016/j.mayocp.2019.01.016

[38] Kloppenborg, R.P., Nederkoorn, P.J., Geerlings, M.I., and van den Berg, E. (2014). Presence and progression of white matter hyperintensities and cognition: a meta-analysis. Neurology 82, 2127–2138 24814849 10.1212/WNL.0000000000000505

[39] van den Berg, E., Geerlings, M.I., Biessels, G.J., Nederkoorn, P.J., and Kloppenborg, R.P. (2018). White matter hyperintensities and cognition in mild cognitive impairment and Alzheimer's disease: a domain-specific meta-analysis. J. Alzheimers Dis. 63, 515–527 29630548 10.3233/JAD-170573

[40] Setnik, L., and Bazarian, J.J. (2007). The characteristics of patients who do not seek medical treatment for traumatic brain injury. Brain Inj. 21, 1–9 17364514 10.1080/02699050601111419

[41] Bogner, J., and Corrigan, J.D. (2009). Reliability and predictive validity of the Ohio State University TBI identification method with prisoners. J. Head Trauma Rehabil. 24, 279–291 19625867 10.1097/HTR.0b013e3181a66356

[42] Corrigan, J.D., and Bogner, J. (2007). Initial reliability and validity of the Ohio State University TBI identification method. J. Head Trauma Rehabil. 22, 318–329 18025964 10.1097/01.HTR.0000300227.67748.77

[43] Jurick, S.M., Crocker, L.D., Keller, A.V., Hoffman, S.N., Bomyea, J., Jacobson, M.W., and Jak, A.J. (2018). The Minnesota Multiphasic Personality Inventory-2-RF in treatment-seeking veterans with history of mild traumatic brain injury. Arch. Clin. Neuropsychol. 34, 366–380 29850866 10.1093/arclin/acy048

[44] Lippa, S.M. (2018). Performance validity testing in neuropsychology: a clinical guide, critical review, and update on a rapidly evolving literature. Clin. Neuropsychol. 32, 391–421 29182444 10.1080/13854046.2017.1406146

